# Morbidity profile of steel pipe production workers

**DOI:** 10.4103/0019-5278.43267

**Published:** 2008-08

**Authors:** Kirti Pandit, Rajnarayan R. Tiwari

**Affiliations:** Occupational Medicine Division, National Institute of Occupational Health, Meghani Nagar, Ahmedabad-380 016, India

**Keywords:** Gujarat, health profile, steel pipe, welding

## Abstract

**Objective:**

To study the different morbid conditions among steel pipe producing workers.

**Methods:**

The present cross-sectional study has been carried out among the workers of one of the steel pipes and tubes manufacturing factory of Gujarat. Hundred workers from the four major departments of the steel pipe production plant, namely welding, pressing machine, X-ray welding and loading/transportation department were covered. The information regarding demographic, occupational, clinical characteristics and diagnosis were recorded on a pre-designed proforma. Statistical analysis included calculation of percentages and proportions and was carried out using the statistical software Epi Info Version 3.3.2.

**Results:**

The mean age of the study subjects was found to be 38.7±7.1 years. The mean duration of exposure was found to be 9.0±3.4 years. Forty-four percent of the subjects had an upper respiratory tract infection, as evidenced by symptoms like dry cough, cough with rhinitis and cough with fever. Symptoms suggestive of allergic bronchitis were observed in 12% of the subjects while symptoms suggestive of heat stress such as prickly heat, dehydration, perspiration and pyrexia were observed in 13% of the subjects.

## INTRODUCTION

India's rapid economic growth is being built on a frame of steel. A soaring demand by sectors like infrastructure, real estate and automobiles, at home and abroad, has put India's steel industry on the world map. India ranked eighth in 2003 in terms of the world crude steel production, with a production figure of 33 MT of crude steel.[1] The large amount of steel produced is then used for making products like pipes, sheets, etc. in different companies.

The process of manufacturing ferroalloys and stainless steel pipes and tubes include many stages. Firstly, roller sheets are cut by PUG machines longitudinally and vertically to give an appropriate shape and size to the sheet. In cold farming, by applying hydraulic pressure to the thick sheet, it is made circular in shape. Certain markers are present on the plate on which pressure is applied by pinches under rolling operation. A pressure drop is applied at that moment, which is dependent on the thickness of the pipe. Then, it is subjected to tuck welding at a distance of 1’ to join the remaining gaps. Under sizing operation, the pipes are subjected to further pressure from above to give them a more circular shape. Inside welding then takes place by using long booms. Electric welding is normally used. Grinding of the edges of the pipes is also done to correct irregularity, if any exists. Finally, the welding process is verified by an X-ray picture. Three more methods are also applied for this final verification. These include hydro testing, ultrasonic testing and real-time testing.

The various processes carried out in the production of steel pipes expose the workers to physical factors like heat, ionizing radiation and noise.[2] Chemical factors like welding fumes containing manganese[3,4] and other heavy metals[5,6] may result in adverse health effects on the neurological,[5,7] respiratory[8–11] and cardiovascular systems.[12] With this background, the present study has been carried out to study the different morbid conditions among steel pipe producing workers.

## MATERIALS AND METHODS

The present cross-sectional study has been carried out among the workers of one of the steel pipe and tube manufacturing factories of Gujarat. The subjects were selected from the four major departments of the steel pipe production plant, namely welding, pressing machine, X-ray welding and loading/transportation departments. In all, 100 subjects were covered.

The information regarding demographic, occupational, clinical characteristics and diagnosis were recorded on a pre-designed proforma. Diagnosis was made purely on the clinical acumen of the treating physician.

Statistical analysis included calculation of percentages and proportions and was carried out using the statistical software Epi Info Version 3.3.2.

## RESULTS

Table 1 describes the distribution of study subjects according to demographic and occupational characteristics. A majority of the study subjects (30%) were in the age group of 35-40 years followed by 19% in the 30-35 years age group. The youngest subject was 23 years of age while the oldest was 57 years of age. The mean age of the study subjects was found to be 38.7±7.1 years. Fifty-one (51%) of the workers were working in this occupation for more 5-10 years while only 17 (17%) workers were working for <5 years. The mean duration of exposure was found to be 9.0±3.4 years. Most of the workers were working for 8–10 hours per day. It was found that only one quarter of the workers were using any form of personal protective device.

**Table 1:** Distribution of study subjects according to demographic and occupational characteristics

Characteristics	Number	Percentage
Age group		
<30	18	18
30–35	19	19
35–40	30	30
40–45	16	16
≥45	17	17
Duration of exposure		
<5	17	17.0
5–10	51	51.0
≥10	32	32.0
Daily working hours		
<8	17	17.0
8–10	70	70.0
≥10	13	13.0
Use of different PPE*		
Boots	10	10.0
Caps or helmets	2	2.0
Gloves	16	16.0
Gloves plus boots	10	10.0
Eye shield	25	25.0

* Includes those using PPE. Multiple responses. PPE, personal protective equipment.

[Table 2] describes the morbid condition among the study subjects according to the departments in which they work. It can be observed from the table that 44% of the subjects had an upper respiratory tract infection, as evidenced by the symptoms like dry cough, cough with rhinitis and cough with fever. Symptoms suggestive of allergic bronchitis were observed in 12% of the subjects while symptoms suggestive of heat stress, such as prickly heat, dehydration, perspiration and pyrexia, were observed in 13% of the subjects. Upper respiratory conditions and allergic bronchitis were common in the welding department while injury was common in the loading and press machine department. The exposure to ionizing radiation was present in the subjects working in the welding X-ray department. Heat stress was found mainly in the subjects involved in the loading and transport of material.

**Table 2:** Distribution of morbid conditions according to various departments

Morbid conditions	Department				Total
		
	Loading/ transport	Press machine	Welding	Welding X-ray
Allergic bronchitis	–	4 (33.3)	5 (41.7)	3 (25)	12
Allergic dermatitis	1 (50)	–	–	1 (50)	2
Back pain	2 (100)	–	–	–	2
Burn	–	–	4 (100)	–	4
Fever	3 (42.8)	2 (28.6)	1 (14.3)	1 (14.3)	7
Heat stress	9 (69.2)	–	–	4 (30.8)	13
Injury	6 (42.9)	8 (57.1)	–	–	14
Upper Respiratory Infection	1 (0.2)	14 (32)	16 (36.3)	13 (29.5)	44
Anorexia	2 (67)	–	–	1 (33)	3
Others	2 (50)	–	–	2 (50)	4
Total	25 (25)	25 (25)	25 (25)	25 (25)	100

Figures in the parentheses are in percentage

## DISCUSSION

In the present study, the workers working in one of the steel pipe production units in Gujarat were considered. In all, the records of 100 subjects were analyzed for the present study. The mean age of the study subjects was found to be 38.7±7.1 years. The mean duration of exposure was found to be 9.0±3.4 years. Most of the workers were working for 8–10 hours per day.

It was found that only one quarter of the workers were using any form of personal protective device. This is a harmful practice as these workers are exposed to welding fumes, ionizing radiation and heat by virtue of their occupation. The non-use of personal protective equipment (PPE) will further aggravate the exposure to deleterious physical agents. The company provides the PPE but the only thing that is needed is the motivation of the workers to use these PPEs. This can be achieved through regular health education of the subjects.

Forty-four percent of the subjects had an upper respiratory tract infection, as evidenced by the symptoms like dry cough, cough with rhinitis and cough with fever. Symptoms suggestive of allergic bronchitis were observed in 12% of the subjects while symptoms suggestive of heat stress, such as prickly heat, dehydration, perspiration and pyrexia, were observed in 13% of the subjects.

Upper respiratory conditions and allergic bronchitis were common in the welding department while injury was common in the loading and press machine department. Earlier studies have reported spirometric changes[8,9] and chronic bronchitis[11] in those exposed to welding fumes. The exposure to ionizing radiation was present in the subjects working in the welding X-ray department. Heat stress was found mainly in the subjects involved in the loading and transport of material. This may be attributed to the exposure to welding fumes in the welding department while the workers in the loading and transport department have to work under extreme climatic conditions and their work is rigorous in nature. Similarly, the process in the press machine department exposes the workers to injuries.

Thus, the present study among the steel ferroalloy product manufacturing workers suggest that they are also exposed to health hazards that are similar to those working in iron foundries or steel production plants. However, before taking a policy decision on the contents of periodic medical examination of these workers, a larger study with a larger sample size should be undertaken.
